# *NUDT15* Genetic Variants in Chinese Han, Uighur, Kirghiz, and Dai Nationalities

**DOI:** 10.3389/fped.2022.832363

**Published:** 2022-04-14

**Authors:** Fang Zhang, Gulbanur Amat, Yanjing Tang, Ru Chen, Xin Tian, Wenting Hu, Changcheng Chen, Shuhong Shen, Yangyang Xie

**Affiliations:** ^1^Department of Hematology/Oncology, Shanghai Children’s Medical Center, Shanghai Jiao Tong University School of Medicine, Shanghai, China; ^2^Changxing Branch of Xinhua Hospital, Shanghai Jiao Tong University School of Medicine, Shanghai, China; ^3^Suzhou University Affiliated Children’s Hospital, Suzhou, China; ^4^Department of Hematology, The Affiliated Children’s Hospital of Kunming Medical University, Kunming Medical University, Kunming, China

**Keywords:** *NUDT15* polymorphism, Uighur, Kirghiz, Dai, mercaptopurine, hematotoxicity

## Abstract

**Background:**

Thiopurines are widely used as anti-cancer and immunosuppressant agents, but have a narrow therapeutic index owing to frequent toxicity and life-threatening bone marrow suppression. The *nudix hydrolase 15* (*NUDT15*) genetic polymorphism is strongly associated with the tolerance and myelosuppressive effect of mercaptopurine administration, but the frequency of *NUDT15* variants is known to vary among different ethnic groups or nationalities. At present, the *NUDT15* gene polymorphism in ethnic minorities such as the Uighur, Kirghiz, and Dai nationalities in China is unclear.

**Procedure:**

DNA samples were isolated from 1,071 Chinese children, including 675 Han children with acute lymphoblastic leukemia and 396 healthy minority children, including 118 Uighur, 126 Kirghiz, and 152 Dai participants. The coding regions of *NUDT15* exons 1 to 3 were amplified by polymerase chain reaction. *NUDT15* genotypes were identified by Sanger sequencing.

**Results:**

Five *NUDT15* genetic variants of coding regions including rs746071566 (c.55_56insGAGTCG), rs186364861 (c.52G > A), c.137C > G, and c.138T > G in exon 1, and the variant rs116855232 (c.415C > T) in exon 3 were found among the participants. The frequency of *NUDT15* rs746071566 variants was lower in the Uighur and Kirghiz populations than in the Han population and in other East Asian nationalities, while the frequency of c.415C > T variants was lower in the Dai population. The c.52G > A variant was relatively uncommon in children of the Han, Uighur, Kirghiz, and Dai ethnic groups. Notably, the rare variants c.137C > G and c.138T > G in a Uighur child were predicted to be disruptive sites.

**Conclusion:**

In summary, our results illustrate the *NUDT15* polymorphisms in Chinese children of Han, Uighur, Kirghiz, and Dai nationalities, and provide the most effective detection recommendations for different ethnic groups to predict thiopurine-related toxicity, which could be used to guide future clinical thiopurine dose adjustment.

## Introduction

Mercaptopurine is widely used as a chemotherapeutic medication for acute lymphoblastic leukemia (ALL) and an immunosuppressive agent for the treatment of inflammatory bowel disease (IBD) (1–4). In the maintenance phase of childhood ALL, mercaptopurine is used as a first-line drug for 2–3 years in modern treatment regimens (5). However, mercaptopurine can cause severe toxicities, especially leukopenia, leading to frequent treatment interruptions, increasing the risk of relapse and fatal infections (6–8). In IBD, thiopurines are also commonly used to preserve the potential of steroids and maintain remission, but up to 40% of patients have to stop treatment due to adverse effects such as hematopoietic toxicity, which leads to subsequent disease recurrence (9, 10). Therefore, there is an urgent need to accurately guide the use of such drugs to reduce adverse reactions and ensure therapeutic effects.

Mercaptopurine is a prodrug that is converted into the active intermediate metabolite thioguanosine triphosphate (TGTP) through a variety of enzymatic reactions. TGTP is further reduced to deoxythioguanosine triphosphate (TdGTP), which binds to DNA during replication to produce DNA-TG, leading to DNA strand breaks, chromosome damage, and ultimately apoptosis. A variety of metabolic enzymes affect the production of mercaptopurine-active products and are closely related to the toxic response (11).

Thiopurine methyltransferase (TPMT) is a key enzyme in the metabolism of mercaptopurine, and has been established to be associated with mercaptopurine toxicity. *TPMT* genotype-guided mercaptopurine therapy has been incorporated into treatment regimens in some European countries (12). However, the frequency of *TPMT* variants is only 2% or less in Asians, which cannot fully explain the high incidence of mercaptopurine-related toxicity in these populations (13, 14).

Recent studies have shown that *nudix hydrolase 15* (*NUDT15*) polymorphisms have a higher mutation frequency in Asian populations compared to European and African populations (15, 16) and is closely associated with thiopurine-related myelosuppression in children with ALL and IBD (17–19). Defective TGTP degradation mediated by the low enzymatic activity of *NUDT15* results in more TGTP available for incorporation into DNA to generate DNA-TG, leading to DNA strand breaks and apoptosis, and thus mitigating toxicity (20). The most common *NUDT15* variants are c.415C > T, c.416G > A, c.52G > A, and c.55_56insGAGTCG, which have been reported to be associated with mercaptopurine-induced myelotoxicity (8, 21, 22). Of these, the *NUDT15* c.415C > T variant has been strongly associated with thiopurine-induced leukopenia, and individuals carrying this homozygous variant are exceptionally sensitive to mercaptopurine and tolerate only 8% of the standard dose (17, 19). Therefore, *NUDT15* genetic variation is the main genetic cause of thiopurine toxicity in Asian populations, while the frequency of *NUDT15* mutations differs among ethnic populations. In Asia, China has 56 ethnic groups, and the total population of the Uighur, Kirghiz, and Dai groups is almost 10 million.

In this study, we investigated the polymorphisms of *NUDT15* in healthy children from three ethnic minorities, including the Uighur, Kirghiz, and Dai ethnic groups and Han Chinese children with ALL, and analyzed their allele frequencies to provide a reference for the individualized dosing of clinical mercaptopurine drugs.

## Materials and Methods

### Study Participants

A total of 1,071 Chinese children were enrolled in this study, of which 675 were Han children with ALL, and 396 were healthy children from three ethnic minorities. The Han children with ALL were treated with the ALL-SCMC-2005 modified protocol at Shanghai Children’s Medical Center from May 2009 to February 2014. In order to compare with ethnic minorities, 118 Uighur (male: 61, female: 57), 126 Kirghiz (male: 51, female: 75), and 152 Dai (male: 88, female: 64) healthy participants younger than 18 years of age were included in the study. Samples of the remaining peripheral blood from healthy ethnic minority children were provided by Kunming Children’s Hospital. We determined the ethnicity of children and their parents by using their registered household registration information, and the ethnicity of previous generations by asking their parents to confirm the three generations of children involved are from the same ethnic group. We collected information on the participants’ name, nationality, sex, and age, and this study was approved by the Ethics Committee of the Shanghai Children’s Medical Center (SCMCIRB – K2020078-1).

### *Nudix Hydrolase 15* Genotyping

Bone marrow karyocytes from Han ALL children in the remission phase and peripheral blood samples from ethnic minority participants were collected. Genomic DNA was extracted from the frozen samples of participants using the TIANamp DNA Kit (Tiangen, Beijing, China), following the manufacturer’s instructions. The quantity and quality of the extracted DNA were assessed using a Nanodrop 8000 (Thermo Fisher Scientific, United States). The three coding sequences (exon 1 to exon 3) of *NUDT15* were amplified by polymerase chain reaction (PCR) and sequenced by Sanger sequencing. For PCR, the forward primer for exon 1 was 5′-CAAAGCACAACTGTAAGCGACT-3′ and the reverse primer was 5′-GAAAGACCCAGCTAGCAAAGAC-3′. The forward and reverse primers for exon 2 were 5′-CGGCCTTCC AAAAGATTACA-3′ and 5′-TGATCTAATCACCTCCCAAGG-3′. The primer sequences for exon 3 were 5′-GCATAGCCTTTGTAAACTGGGC-3′ in the forward direction and 5′-CTCACTGGAAAAAGATTCCTTAGC-3′ in the reverse direction. Each 50 μL PCR mixture contained 25 μL 2X Taq PCR Master Mix (with dye, Sangon Biotech, Shanghai, China), 20 μM of each primer, and 200 ng of genomic DNA; and ddH2O was added to 50 μL. PCR for exon 1 and exon 2 was performed by heating to 94°C for 1 min, followed by 30 cycles at 94°C for 30 s, maintenance at 53°C for 30 s, and then 72°C for 1 min; with the final extension performed at 72°C for 5 min. PCR for exon 3 was performed with a starting temperature of 94°C for 1 min, followed by 35 cycles of 30 s at 94°C, 1 min at 55°C, and 3 min at 72°C, with a final extension at 72°C for 5 min. The PCR products were separated by 2% agarose gel electrophoresis, and the target bands were extracted using a Gel Extraction Kit D2500 (Omega, Guangzhou, China). The variants shown in this article correspond to version NM_018283.4.

### Statistical Analysis

The *NUDT15* coding regions of exons 1 to 3 were determined by direct sequencing, and the sequencing data were analyzed using SnapGene software (GSL Biotech, Chicago, Illinois, United States). The Pearson chi-square test was used to verify that the *NUDT15* c.52G > A, c.415C > T distributions in the three ethnic minority populations were consistent with the Hardy-Weinberg equilibrium law. Allele frequency was calculated as:


$$\mathrm{allele}\,\mathrm{frequency}=\frac{\mathrm{total}\,\mathrm{number}\,\mathrm{of}\,\mathrm{heterozygotes}+\left(\mathrm{total}\,\mathrm{number}\,\mathrm{of}\,\mathrm{homozygotes}\times 2\right)}{\mathrm{total}\,\mathrm{sample}\,\mathrm{size}\times 2}\times 100\%$$

The chi-square test and Fisher exact test with an R × C column was used to analyze differences in distribution between ethnic minorities and other populations.

## Results

### Characteristics of Study Participants

*NUDT15* genotyping was conducted for a total of 1071 Chinese children with 675 Han Chinese children diagnosed with ALL and 396 healthy children from ethnic minorities, including 118 children of Uighur nationality (male: female = 1.07: 1), 126 Kirghiz children (male: female = 0.68: 1), and 152 Dai children (male: female = 1.38: 1) (Table 1). They all belonged to families of the same ethnicity, with at least three generations. The median ages of the Han, Uighur, Kirghiz, and Dai children were 5, 5, 7, and 5 years, respectively.

**TABLE 1:** Characteristics of the Han nationality and three ethnic minorities in our cohort.

Variable	Han (*N* = 675)	Uighur (*N* = 118)	Kirghiz (*N* = 126)	Dai (*N* = 152)
**Age (years)**				
≤1	17	9	3	11
1 to 10	549	83	94	128
≥10	109	26	29	13
**Gender**				
Male	410	61	51	88
Female	265	57	75	64

### *Nudix Hydrolase 15* Genotypes in Han Chinese Children and Healthy Uighur, Kirghiz, and Dai Children

**FIGURE 1:**
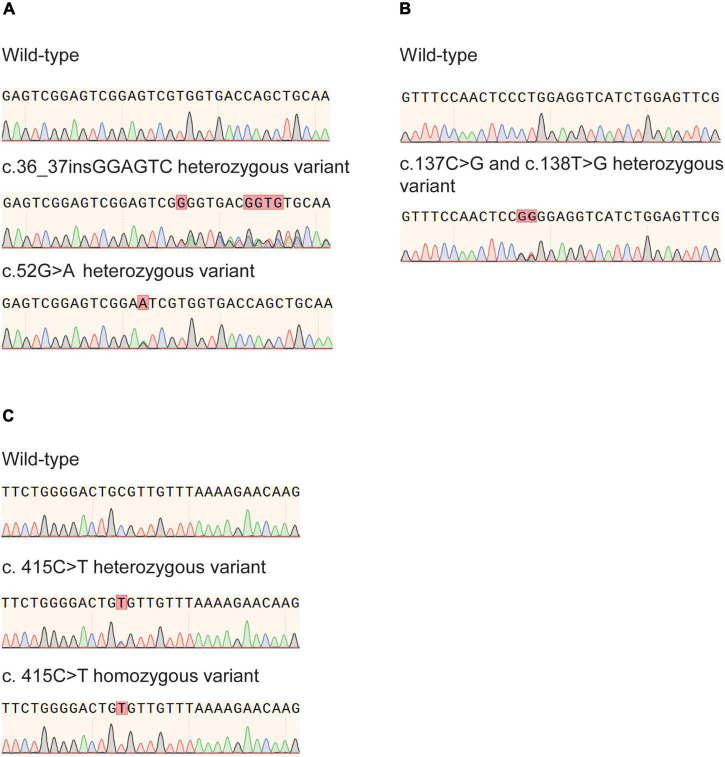
*Nudix hydrolase 15 (NUDT15)* gene variants identified by Sanger sequencing. **(A)** c.55_56insGAGTCG and c.52G>A heterozygous variant in exon 1. **(B)** Rare heterozygous variants c.137C>G and c.138T > G in exon 1 were confirmed. **(C)** c. 415C > T heterozygous and homozygous variant in exon 3.

In the present study, the previously reported variants rs746071566 (c.55_56insGAGTCG) and rs186364861 (c.52G > A) in exon 1 and the variant rs116855232 (c.415C > T) in exon 3 were confirmed (Figures 1A,C). Genotyping results are presented in Table 2. In all three minority populations, *NUDT15* c.415C > T was the most common variant type, followed by c.55_56insGAGTCG and c.52G > A. Our results showed that 29.9% (*n* = 202) of Han children with ALL carried the *NUDT15* gene with one or more variants. The proportion of healthy children carrying variants in the *NUDT15* gene was 16.1% (*n* = 19) (*P* = 0.003), 19.0% (*n* = 24) (*P* = 0.017), and 16.4% (*n* = 25) (*P* = 0.001) for the Uighur, Kirghiz, and Dai ethnic groups compared to Han children, respectively.

**TABLE 2:** Distribution of *nudix hydrolase 15 (NUDT15)* variants among Han and ethnic minority populations.

Reference SNP Id	Genetic variant	Han (*N* = 675)				Uighur (*N* = 118)				Kirghiz (*N* = 126)				Dai (*N* = 152)			
					
		−⁣/⁣−	−⁣/⁣ +	+ ⁣/⁣ +	MAF	−⁣/⁣−	−⁣/⁣ +	+ ⁣/⁣ +	MAF	−⁣/⁣−	−⁣/⁣ +	+ ⁣/⁣ +	MAF	−⁣/⁣−	−⁣/⁣ +	+ ⁣/⁣ +	MAF
rs746071566	c.55_56insGAGTCG	588	87	0	6.4%	114	4	0	1.7%	125	1	0	0.4%	140	12	0	3.9%
rs186364861	c.52G > A	656	19	0	1.4%	118	0	0	0	125	1	0	0.4%	150	2	0	0.7%
rs116855232	c.415C > T	511	150	14	13.2%	101	16	1	7.6%	103	23	0	9.1%	135	17	0	5.6%

*SNP, single nucleotide polymorphism; MAF, minor allele frequency.*

We described the results of *NUDT15* gene polymorphism in Han Chinese children with ALL, showing the minor allele frequencies (MAFs) of 6.4%, 1.4%, and 13.2% for the *NUDT15* c.55_56insGAGTCG, c.52G > A and c.415C > T variants, respectively. According to *NUDT15* genotyping in 118 children of Uighur nationality, 99 children were wild-type (83.9%), and MAFs of *NUDT15* c.55_56insGAGTCG and c.415C > T were 1.7% and 7.6%, respectively (Table 2). In addition, 13.6% Uighur children were heterozygous for *NUDT15* c.415C > T, and 0.8% were homozygous variants. However, the *NUDT15* c.52G > A variant was not found in the Uighur participants. In the current study of 126 Kirghiz children, the MAF of c.55_56insGAGTCG was 0.4%, while 9.1% for *NUDT15* c.415C > T. Only one Kirghiz child with a variant of c.52G > A was observed. Among 152 Dai children, the highest variation frequency was c.55_56insGAGTCG, with an MAF of 3.9%; however, the c.415C > T locus had the lowest MAF of 5.6% compared to the other two minorities. Moreover, two Dai children were identified carrying heterozygous c.52G > A variants.

**FIGURE 2:**
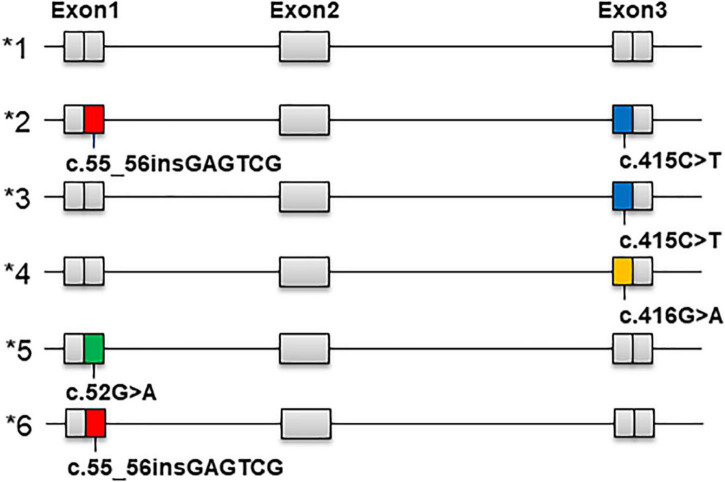
Nomenclature of *NUDT15* star alleles (*1 to *6), corresponding to the variant version NM_018283.4.

The star allele nomenclature in Figure 2 is derived from PharmVar.^1^ We calculated the frequencies of each star allele and compared them with those published by PharmVar (Table 3). Among the four ethnic groups in this study and the Central/South Asian, East Asian, European and Latin populations in PharmVar, the most common star allele was *1 (wild-type), whose frequencies were 83.8%, 91.5%, 90.5% and 91.8% in Han, Uighur, Kirghiz and Dai populations, respectively, and higher than 85% in the Central/South Asian, East Asian, European and Latin populations. Regarding the star alleles carrying variants, *3 was the most common, and its frequency was highest in the Kirghiz population (8.7%), followed by Han (8.1%), Central/South Asian (6.7%), Uighur (6.4%), East Asian (6.1%) and Dai (3.6%). The frequency of *2 was highest for Han Chinese at 5%, with East Asian and Latino populations at close to 3.5% and under 2% for Uighur, Kirghiz and Dai. The frequencies of all other star alleles were lower. In addition, we also compared diplotype frequencies in different populations (Table 4) and the most common variant diplotype was *1/*3, with the frequency of *1/*3 in the four ethnic groups in this study being closer to that of Central/South and East Asian and much higher than that of European and Latino populations.

**TABLE 3:** *Nudix hydrolase 15* allele Frequencies in biogeographic populations.

*NUDT15* Allele	Han (*N* = 675)	Uighur (*N* = 118)	Kirghiz (*N* = 126)	Dai (*N* = 152)	Central/South Asian	East Asian	European	Latino
*1	83.8%	91.5%	90.5%	91.8%	93.0%	87.9%	99.3%	93.6%
*2	5.0%	1.3%	0.4%	2.0%	0	3.5%	0	3.7%
*3	8.1%	6.4%	8.7%	3.6%	6.7%	6.1%	0.2%	0.8%
*4	0.1%	0	0	0	0	0.1%	0	1.8%
*5	1.4%	0	0.4%	0.7%	0	1.1%	0	0
*6	1.4%	0.4%	0	2.0%	0.2%	1.3%	0.3%	0.2%

*Data for East Asian European Latinos in Central/South Asia are derived from PharmVar.*

**TABLE 4:** *Nudix hydrolase 15* Diplotype frequencies in biogeographical groups.

*NUDT15* Diplotype	Han (*N* = 675)	Uighur (*N* = 118)	Kirghiz (*N* = 126)	Dai (*N* = 152)	Central/South Asian	East Asian	European	Latino
*1/*1	69.6%	83.9%	81.0%	83.6%	86.5%	77.2%	98.6%	87.7%
*1/*2	8.7%	2.5%	0.8%	3.9%	0	6.2%	0	6.8%
*1/*3	13.0%	11.0%	17.5%	7.2%	12.5%	10.6%	0.4%	1.4%
*1/*4	0.1%	0	0	0	0	0.2%	0	3.3%
*1/*5	2.2%	0	0.8%	1.3%	0.1%	2.0%	0	0
*1/*6	2.7%	0.8%	0	3.9%	0.4%	2.3%	0.6%	0.3%
*2/*2	0	0	0	0	0	0.1%	0	0.1%
*2/*3	1.3%	0	0	0	0	0.4%	0	0.1%
*2/*4	0	0	0	0	0	0	0	0.1%
*2/*5	0	0	0	0	0	0.1%	0	0
*2/*6	0	0	0	0	0	0.1%	0	0
*3/*3	0.7%	0.8%	0	0	0.4%	0.4%	0	0
*3/*5	0.4%	0	0	0	0	0.1%	0	0
*3/*6	0	0	0	0	0	0.2%	0	0
*5/*6	0.1%	0	0	0	0	0	0	0

*Data for East Asian European Latinos in Central/South Asia are derived from PharmVar.*

The Hardy Weinberg genetic equilibrium test for genotypes at the c.415C > T and c.52G > A sites in the Han, Uighur, Kirghiz, and Dai ethnic groups, showed that the distribution of both loci was consistent with Hardy Weinberg genetic equilibrium (*P* > 0.05), indicating that the selected samples were from the same Mendelian population and could reflect the distribution of c.415C > T and c.52G > A in the target population.

### Comparison Between Han and Ethnic Minorities With Other East Asian Populations

**FIGURE 3:**
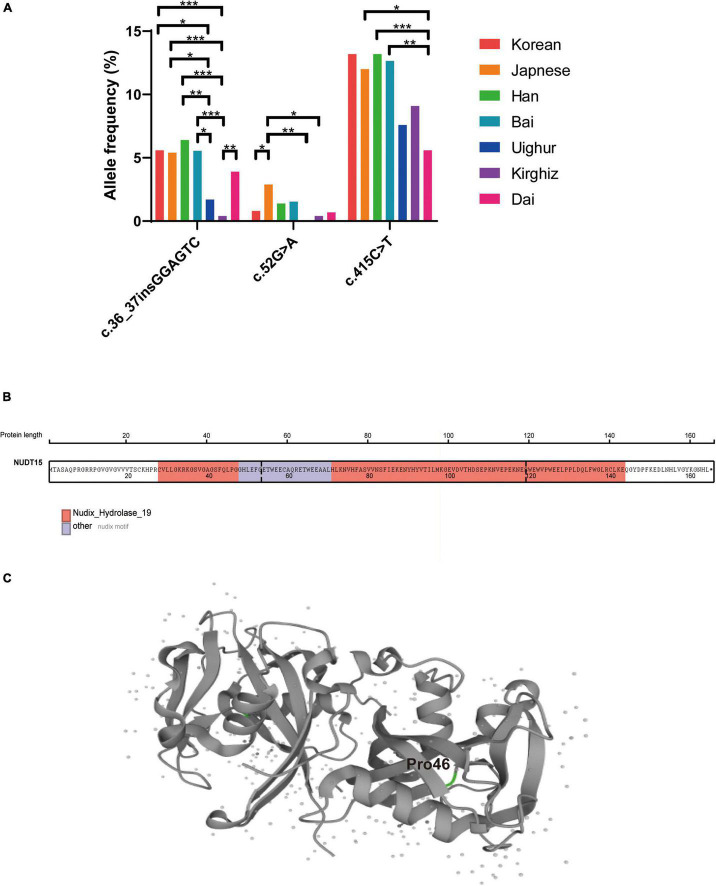
Comparison of the frequencies of variation at multiple sites of *NUDT15* in different ethnic groups and the *NUDT15* structural domain and protein structure. **(A)** *P < 0.05; **P < 0.01; ***P < 0.001. **(B)** The *NUDT15* structural domain obtained from St. Jude Cloud is marked in color, and p.P46R (c.137C > G and c.138T > G) was located on the Nudix-Hydrolase-19 functional domain. **(C)** The position of p.P46R in the NUDT15 protein derived from Uniprot is highlighted in green.

To clarify the distribution of *NUDT15* variants in Chinese ethnic minority populations, we conducted a two-by-two comparison of the c.55_56insGAGTCG, c.52G > A, and c.415C > T variant frequencies in seven ethnic groups: Korean (23), Japanese (24), Chinese Han, Bai (25), Uighur, Kirghiz, and Dai, of which Bai is also an ethnic minority in China. The allele frequency of *NUDT15* c.55_56insGAGTCG was 5.6% in Korea (23), 5.4% in Japan (24), 6.4% in Chinese Han, and 5.6% in Chinese Bai (25). We found that the c.55_56insGAGTCG variant was significantly less frequent in the Uighur and Kirghiz ethnic groups than in the other ethnic groups, and the frequency of variation at this site in the Dai ethnic group was not significantly different from that of the other ethnic groups. The *NUDT15* c.52G > A allele frequency was below 2% for all ethnic groups except Japan (2.9%) (24) (Figure 3A). The MAFs of c.52G > A variant were lower in children of Han, Uighur, Kirghiz and Dai ethnic groups.

The single nucleotide polymorphism (SNP) c.415C > T is the most important variant of *NUDT15* related to thiopurine toxicity, which leads to almost complete loss of enzyme activity and protein stability *in vitro* and induces severe bone marrow suppression. It has been reported that the allele frequency of the *NUDT15* c.415C > T variant is 13.2% in South Korea (23), 12.0% in Japan (24), and 12.7% in the Bai nationality in China (25). The distribution of *NUDT15* c.415C > T variants in our results for the Han, Uighur and Kirghiz ethnic groups was similar to that for other ethnic groups, except for the Dai nationality group. The frequency of *NUDT15* c.415C > T variants was significantly lower in the Dai population than in the Japanese (*P* = 0.02), Han populations (*P* = 0.0007), and Bai populations (*P* = 0.004).

### Rare Variants in Han and Minorities Children

We identified eight rare heterozygous variants in Han and minority children and showed the protein abundance score, drug sensitivity score and final classification referred to a previous article by Jun J Yang (26). There were four variants (c.37G > A, c.137C > G, c.138T > G, c.154T > C) in exon 1, three variants (c.202G > A, c.254T > C and c.272A > C) in exon 2, and one variant (c.422T > G) in exon 3 (Table 5). Remarkably, c.137C > G and c.138T > G heterozygous mutations at adjacent loci were found in one Uighur child (Figure 1B). This missense mutation resulted in a change of amino acid 46 from proline to arginine (P46R). To explore the impact of this variation, we revealed that this variant site was located in the Nudix-Hydrolase-19 functional domain (Figure 3B), which is a structural part of the beta fold in the protein (Figure 3C).

**TABLE 5:** Rare *NUDT15* variants among Han and ethnic minority populations.

Genetic variant	Protein alteration	Exon	Ethnicity	Protein abundance score (26)	Drug sensitivity score (26)	Final classification (26)
c.37G > A	G13R	exon1	Han	0.78	1.27	wt-like
c.137C > G and c.138T > G	P46R	exon1	Uighur	0.14	0.04	damaging
c.154T > C	F52L	exon1	Han	/	1.07	/
c.202G > A	A68T	exon2	Han	1.06	1.00	wt-like
c.254T > C	I85T	exon2	Han	1.09	1.23	wt-like
c.272A > C	H91P	exon2	Han	0.49	0.62	wt-like
c.422T > G	L141*	exon3	Han	/	/	/

## Discussion

Mercaptopurine is a cornerstone drug for ALL and IBD, but is limited by the adverse effects such as myelosuppression, alopecia, hepatotoxicity, and pancreatitis (27). Leukopenia is the most common myelosuppressive manifestation of thiopurines, occurring in 15.36% of IBD patients (28). Severe myelosuppression can be life-threatening when treating ALL with 6-MP (6-mercaptopurine), and interruption of mercaptopurine therapy due to induced toxicity can increase the risk of relapse. Therefore special caution must be exercised toward mercaptopurine effects on myelotoxicity (29, 30).

*NUDT15* germline variants are closely associated with mercaptopurine-induced hematotoxicity and vary considerably in their distribution across ethnic groups (31, 32), with the risk allele of *NUDT15* c.415C > T being the most common among East Asians (9.8%), very rare in Europe (0.2%), and not observed in Africa (19). Among the 56 ethnic groups in China, only the Bai ethnic groups of the Yunnan and Han nationality have been studied for the distribution of the *NUDT15* polymorphism (25, 33). This study investigated the distribution of *NUDT15* gene polymorphisms among healthy Uighur and Kirghiz children in Xinjiang and Dai children in Yunnan, China. Each of these minorities has a population of over one million. We also conducted a large sample study on the distribution of *NUDT15* genotypes in Han Chinese children with ALL. The *NUDT15* gene encodes an enzyme that negatively regulates the activation and toxicity of thiopurine and has no effect on the pathogenesis of leukemia. Therefore, this cohort of children with ALL can be considered as a reference for the normal Han population. The frequencies of children carrying the *NUDT15* variants were 29.9%, 16.1%, 19.0%, and 16.4% in the Han, Uighur, Kirghiz, and Dai ethnic groups in China, respectively. The distribution of both the c.415C > T and c.52G > A variants in the four ethnic groups is consistent with Hardy-Weinberg genetic equilibrium, indicating that the population surveyed is in genetic equilibrium and that the data from this population survey are reliable.

Numerous studies have reported increased susceptibility to mercaptopurine toxicity with the *NUDT15* variant c.415C > T in patients with ALL (19, 34, 35) and IBD (36–38). Compared to ALL patients with the CC genotype who tolerated 83.5% of the standard dose, patients with the TC and TT genotypes tolerated 63% and 8.3%, respectively (19). The allele frequency of *NUDT15* c.415C > T was 13.2% in Han, 7.6% in Uighur, 9.1% in Kirghiz, and 5.6% in Dai. Previous reports have shown that the allele frequency for *NUDT15* c.415C > T was 16.3% and 11.62% in Japan (18, 35); 11.6% and 15.7% in China (14, 33); and 13.2% in Korea (23). In terms of c.415C > T variant frequency, our report of allele frequency of the Uighurs and Kirghiz is similar to other regional findings, while Dai has significantly lower variant frequencies than the majority East Asian population.

In addition to c.415C > T, a Chinese study of 732 patients with IBD concluded that *NUDT15* c.55_56insGAGTCG and c.52G > A variants are also risk factors for thiopurine-induced leukopenia (21). However, a Japanese study in IBD patients concluded that c.55_56insGAGTCG and c.52G > A variants were not significantly associated with leukopenia. In addition, Moriyama et al. identified that *NUDT15* coding variants (c.415C > T, c.55_56insGAGTCG and c.52G > A) resulted in 74.4–100% loss of nucleotide diphosphatase activity. Therefore, the effect of c.55_56insGAGTCG and c.52G > A variants on mercaptopurine induced toxicity is not yet determined. In our study, the c.55_56insGAGTCG variant was significantly lower in Uighur and Kirghiz children than in Han and other Asian nationalities, and the c.52G > A variant in Uighur, Kirghiz, Dai, and Han nationality was also lower. Since the frequency of *NUDT15* genetic variants varies among different races, our results support that ethnic differences should be considered when using *NUDT15* genotyping. We recommend that it is not necessary to detect these two variants in Uighur and Kirghiz patients and the c.52G > A variant in Dai and Han patients, but the c.415C > T variant in exon 3 must be detected. This helps to use the least economic burden to predict toxicity and guide thiopurine dosing adjustment.

The rare *NUDT15* c.137C > G and c.138T > G heterozygous mutation in this study were detected in a healthy child from Uighur, and these variants resulted in a change in amino acid 46 encoded by *NUDT15* from proline to arginine (p.P46R). This site is located in the functional structural domain of the protein encoded by *NUDT15*. Yang et al. determined the effect of variation in protein abundance and thiopurine cytotoxicity using a massively parallel *NUDT15* functional assay, showing that the p.P46R variant of *NUDT15* had protein abundance and drug sensitivity scores of 0.14 and 0.04, respectively (26). The protein abundance of this variant is lower than that of the known toxicity risk variant Arg139Cys, suggesting that this missense mutation exhibits a significant decrease in the thermostability of the NUDT15 protein, and the drug sensitivity score was also low, indicating low enzymatic activity of the p.P46R variant. Therefore, the p.P46R missense mutation is a disruptive mutation site that affects the structural stability of the enzyme and the activity of NUDT15. We need to further investigate the relationship between patients carrying this disruptive mutant and mercaptopurine dose-related side effects and drug tolerance in Uighur patients with ALL or IBD.

Our study has some limitations. The sample size of this study on three ethnic minorities was relatively small, and it is expected that the sample size will be further expanded in future studies to confirm our results. Subsequent studies should include clinical tolerance and treatment response to mercaptopurine in ethnic minority children with ALL carrying different *NUDT15* genotypes.

In conclusion, this study revealed polymorphisms in the *NUDT15* gene in Han, Uighur, Kirghiz, and Dai children, providing a basis for the clinical use of mercaptopurine in Han Chinese and ethnic minority patients. In order for the clinical test to be most meaningful for Uighur or Kirghiz patients, the test needs to include the c.415C > T variant, and for Dai and Han Chinese the test requires the inclusion of the c.55_56insGAGTCG and c.415C > T variants. The *NUDT15* genetic variants in various ethnic groups may guide future studies in the individualized administration of mercaptopurine drugs in a clinical setting for patients from Han and ethnic minority populations (such as Uighur, Kirghiz, and Dai) in China.

## Data Availability Statement

The original contributions presented in the study are included in the article/supplementary material, and further inquiries can be directed to the corresponding authors.

## Ethics Statement

The studies involving human participants were reviewed and approved by the Ethics Committee of the Shanghai Children’s Medical Center. Written informed consent from the participants’ legal guardian/next of kin was not required to participate in this study in accordance with the national legislation and the institutional requirements.

## Author Contributions

YX and SS conceived the idea of the study. FZ, GA, YT, and RC performed the experiments and analyzed the data. FZ wrote the manuscript. All authors contributed to the article and approved the submitted version.

## Conflict of Interest

The authors declare that the research was conducted in the absence of any commercial or financial relationships that could be construed as a potential conflict of interest.

## Publisher’s Note

All claims expressed in this article are solely those of the authors and do not necessarily represent those of their affiliated organizations, or those of the publisher, the editors and the reviewers. Any product that may be evaluated in this article, or claim that may be made by its manufacturer, is not guaranteed or endorsed by the publisher.

## Footnotes

1
https://www.pharmvar.org/gene/NUDT15
